# Ameliorative Effect of Valeric Acid Against Psychophysiological Chronic Unpredictable Stress

**DOI:** 10.3390/biomedicines14040795

**Published:** 2026-03-31

**Authors:** Bindu Kumari, Gireesh Kumar Singh, Gyan Prakash Modi, Hitesh Harsukhbhai Chandpa, Ravi Bhushan Singh, Geeta Rai, Khushbu Priya, Dhananjay Kumar Singh

**Affiliations:** 1 Department of Pharmacy, Central University of South Bihar, Gaya 824236, Bihar, India; bindu@cusb.ac.in (B.K.); gireesh@cusb.ac.in (G.K.S.); 2 Department of Pharmaceutical Engineering and Technology, Indian Institute of Technology (BHU), Varanasi 221005, Uttar Pradesh, India; gpmodi.phe@iitbhu.ac.in (G.P.M.); hiteshhchandpa.rs.phe22@itbhu.ac.in (H.H.C.); 3 Ravi Bhushan Singh, Centre of Excellence in Pharmaceutical Sciences (CEPS), Guru Gobind Singh Indraprastha University, Dwarka 110078, New Delhi, India; ravimgs@gmail.com; 4 Department of Molecular and Human Genetics, Institute of Science, Banaras Hindu University, Varanasi 221005, India; geetarair74@gmail.com (G.R.); khushbupriya.khushi@gmail.com (K.P.); 5 Veterinary Clinical Sciences, University of Minnesota, Twin Cities, MN 55455, USA

**Keywords:** Psychophysiological stress, valeric acid, corticosterone, IL-6, TNF-α, IL-1β

## Abstract

**Background**: Chronic unpredictable stress triggers various pathological and metabolic alterations by modulating psychophysiological balance. Valeric acid (VA), a postbiotic material, has been reported to mitigate stress-induced behavioral changes in rodents. **Objectives**: To investigate the protective effect of valeric acid against chronic unpredictable stress in a rodent model by assessing neuro-physiological alterations along with changes in biochemical parameters to confirm the possible mechanism. **Methods**: A 14-day chronic unpredictable stress (CUS) model in albino Wistar rats was developed to check the stress-induced changes using forced swim test, tail suspension test and sexual behavior observation. Quantification of IL-6, TNF-α, IL-1β, plasma corticosterone level and oxidative stress parameters were also done. **Results**: Findings revealed the protective effects of valeric acid against CUS, which reversed the depression caused by a forced swim and tail suspension test in rats. Proinflammatory and oxidative stress markers were significantly (*p* < 0.05) restored in CUS rats treated with valeric acid as compared with the vehicle control, which was comparable to the standard drug, *Panax ginseng*. **Conclusions**: The present study concludes that valeric acid demonstrated significant (*p* < 0.05) anti-stress effect by modulating both behavioral responses and stress-related biochemical modifications.

## 1. Introduction

Stress is a nonspecific physiological and social response of the body to immediate or potential threats to homeostasis. It arises when internal or external stimuli challenge the body’s equilibrium, triggering a range of behavioral and physical reactions aimed at adaptation and maintaining internal balance [1,2]. Stressors may be physical or psychological, and the stress response is mediated through a complex interplay among the nervous, endocrine, and immune systems [3]. Stress affects individuals at multiple levels—from cellular to organ systems—altering physiological and metabolic processes [4,5]. At the cellular level, stress exposure can activate survival pathways or initiate programmed cell death to eliminate damaged cells. Major mechanisms through which stressors damage cells include oxidative stress caused by free radicals, DNA damage, and the accumulation of unfolded or misfolded proteins [6]. Chronic exposure to physical, psychological, or environmental stressors has been associated with increased morbidity and mortality [7]. Although the complexity of stress responses presents substantial research challenges, studies in both humans and animals have greatly advanced our understanding [8]. Animal models of stress are still widely used to investigate physiological disturbances that resemble aspects of psychiatric disorders, to screen antidepressant agents, and to explore the neurobiological mechanisms underlying depression [9]. While no single model can fully replicate the complexity of human depression, different experimental paradigms target specific components such as chronic stress exposure, genetic vulnerability, or early-life adversity [10].

The chronic unpredictable stress (CUS) rodent model is most widely used to study long-term psychophysiological stress. This paradigm involves repeated exposure to varied stressors in an unpredictable sequence, leading to physiological and behavioral alterations including metabolic dysregulation, impaired neuroendocrine function, anxiety, and depressive-like behaviors [11]. The pathophysiology of stress involves multiple interacting biological systems. One of the most widely recognized frameworks is the hypothalamic–pituitary–adrenal (HPA) axis, along with the gut–brain axis. The HPA axis, in coordination with the autonomic nervous system and the immune system, plays a central role in mediating the stress response. Upon exposure to stressors, the hypothalamus releases corticotropin releasing hormone (CRH), which stimulates the pituitary gland to secrete adrenocorticotropic hormone (ACTH). ACTH then triggers the adrenal cortex to synthesize and release glucocorticoids (GCs), with cortisol being the primary hormone responsible for metabolic changes during stress [12]. Simultaneously, the gut–brain axis—a bidirectional communication network linking the gut microbiota with the central nervous system—modulates emotional and cognitive functions. Dysbiosis, or imbalance in the gut microbiota, can influence HPA axis activity, promote systemic inflammation, and increase gut permeability. Recent research has focused on identifying naturally occurring bioactive compounds that may protect against stress-induced neurobiological changes [13].

Valeric acid (VA), a postbiotic molecule produced by gut microbiota and found naturally in plants such as *Valeriana officinalis* and *Angelica archangelica*, is also generated through anaerobic microbial fermentation [14]. As a key bioactive component of valerian root, valeric acid has long been used for its sedative and hypnotic properties in the treatment of sleep disorders [14]. Beyond these effects, VA exhibits diverse pharmacological activities, including antihypertensive, antidiabetic, anticancer, immunomodulatory, and anti-inflammatory actions. It also modulates molecular pathways implicated in conditions such as Parkinson’s disease, Alzheimer’s disease, and epilepsy [15]. Recent findings suggest that postbiotics, a product of probiotic fermentation, may modulate biochemical signaling pathways related to oxidative stress, metabolism, inflammation, and immunity. The development of postbiotic-based therapeutics and dietary formulations offers a promising approach for improving stress-related psychophysiological disorders. However, despite its broad pharmacological potential, the role of valeric acid in alleviating psychological stress induced by CUS has not yet been investigated. The present study therefore aims to evaluate the protective effects of valeric by assessing neurophysiological and behavioral outcomes of psychophysiological stress induced by chronic unpredictable stress in a rodent model.

## 2. Material and Methods

All reagents, chemicals and solvents used in this study were of analytical grades and procured from authentic manufacturers and suppliers. Valeric acid (CAS no-109-52-4) was purchased from Tokyo Chemical Industry (TCI chemicals, Japan). Panax ginseng (PG) extract powder was procured from Nutrija life science (Madhya Pradesh, India). IL-1β (Cat No. 670.040.096), IL-6 (Cat No. 670.010.096) and TNF-α (Cat No. 865.000.096) ELISA kits (rats specific) were purchased from Diaclone (Besancon, France) and Corticosterone (Cat No. EELR016) ELISA kit (rat-specific) was purchased from Invitrogen by ThermoFisher Scientific, India.

### 2.1. Animal Subjects

Adult male and female albino Wistar rats weighing 150 ± 10 g were obtained from the Central Animal House at the Indian Institute of Technology, Banaras Hindu University (IIT, BHU), Varanasi, under registration number [2123/GO/Re/s/21/CPCSEA]. The animals were randomly allocated into various experimental groups and maintained under standard laboratory conditions with temperature controlled at 25 ± 1 °C and a 12 h light/dark cycle. Commercial pellet feed and water were provided ad libitum throughout the study period. Prior to experimentation, all rats underwent a one-week acclimatization period to adapt to the laboratory environment. All experimental protocols followed the National Institutes of Health Guide for Care and Use of Laboratory Animals (Publication No. 85-23, revised 1985) and received prior ethical approval from the Institutional Animal Ethics Committee [IIT(BHU)IAEC/2023/II/044] of IIT, BHU, dated 25 August 2023.

### 2.2. Drug Treatment

Animals were classified into five groups (*n* = 6 only male rats in each group) [16,17]. Treatments were given per os for fourteen successive days simultaneously with CUS induction. The treatment schedule for classified groups are as follows: Group I (vehicle control) received 0.3% carboxymethyl cellulose (CMC) suspension; Group II (vehicle) received 0.3% CMC with CUS (two stressors applied per day); Group III (CUS + VA) received VA (50 mg/kg BW; Group IV (CUS + VA) received VA (100 mg/kg BW); and Group V (CUS + PG) received PG 100 mg/kg BW along with daily stress exposure. The treatments were given daily prior to the first CUS exposure [16,18].

### 2.3. Induction of Chronic Unpredictable Stress

The CUS model exposed rats to two different daily stressors for 14 days, as described by Katz, Rasheed and Ahmad, with slight modifications [19,20,21]. To prevent habituation, stressors were randomized with minimal repetition. The stressors include fasting (food deprivation for 20 h), restraint stress (3 h in cylindrical steel tubes), tail pinching (5 min with steel clips), forced swimming (30 min in water-filled jars at 25 °C), overnight wet cage bedding, water deprivation (20 h), isolation (12 h alone), day–night reversal (3 h darkness during day, 12 h bright light at night), and cold-restraint (3 h at 4 °C while restrained). The individual stressors and their duration of exposure during every day of the CUS are summarized in Table 1.

### 2.4. Changes in Body Weight

Rat body weight (BW) was measured initially and daily throughout CUS exposure. Weight variation percentage was calculated using [22]

Weight Variation (%) = [(Final Weight − Initial Weight)/Final Weight] × 100.

### 2.5. Electrocardiography (ECG and HRV)/Hemodynamic Changes

Electrocardiogram (ECG) recording occurred on day 13 post-treatment. Animals were anesthetized using isoflurane (2% in 100% oxygen at 300 mL/min flow rate, following 3–5% isoflurane). Throughout the procedure, body temperature was maintained at 37 ± 1 °C using thermostat-controlled heating pads. Gold-plated platinum electrodes were positioned on the left and right thorax sides (positive/negative) and the peritoneal region (neutral). Electrodes were connected to LabScribe software Version 3.0 through an iWire-B3G ECG module and IXTA data acquisition unit for analog-to-digital signal conversion. Recorded graphs enabled ECG and heart rate variability (HRV) parameter calculations. Hemodynamic analysis began with R wave verification followed by heart rate and ECG parameter calculations. Time and frequency-domain HRV parameters were computed using LabScribe software Version 3.0, consistent with previously established protocols [23,24].

### 2.6. Stress-Induced Blood Glucose Level Assessment

The rats were fasted overnight prior to measured blood glucose level. The rats were anesthetized under the anesthesia chamber with diethyl ether. Blood was taken from the retro-orbital sinus while the rats were anesthetized. The blood glucose level of each rat was measured using a glucometer (MedHub).

### 2.7. Behavioral Perturbation

A behavioral test was performed on day 14 after the final stress exposure and valeric acid administration.

#### 2.7.1. Forced Swimming Test

The methodology outlined by Porsolt in 1978 [25] was used with some modification. In brief, rats were individually placed in glass jars (45 cm height × 25 cm length × 20 cm width) filled to 20 cm water depth at 25 ± 2 °C, ensuring they could not touch the bottom with their feet. Swimming duration was 10 min with immobility recording during the final 5 min. Immobility was defined as complete swimming cessation with head floating above water. Post-testing, rats were dried and placed in heated cages for 15 min before returning to home cages. Blind observers measured immobility duration manually using a stopwatch, representing behavioral despair [25].

#### 2.7.2. Tail Suspension Test

Tail suspension test (TST) was conducted following the protocol outlined by Steru in 1985 [26]. Rats were suspended by adhesive tape approximately 1 cm from tail tips, 30 min after the last drug administration. Distance between the tail tip and floor was maintained at approximately 60 cm. Immobility period (absence of any effort to correct the position) was recorded for 6 min using stopwatches [26].

#### 2.7.3. Light/Dark Transition Test

Using Crawley and Goodwin in 1980 [27] methodology, the light/dark transition test apparatus consisted of 45 cm × 45 cm × 35 cm cages divided into equal compartments with 4 cm × 3 cm rectangular openings. One compartment was brightly illuminated (400 lux), the other dark (10 lux). Rats initially placed in dark compartments could move freely for 10 min. Parameters measured included distance traveled (cm), total number of transitions, latency to enter light compartment (s), and the time spent in the light compartment was recorded [27].

#### 2.7.4. Sexual Behavior

Male rats were individually housed together with 2 estrous female rats (treated with estradiol valerate 5 μg/rat followed by hydroxyprogesterone 1.5 mg/rat subcutaneously after 48 h) in dimly lit rooms for 10 min. Total mounting numbers exhibited by males were recorded [28].

### 2.8. Specimen (Blood) Collection, Processing and Storage

After the last stress regimen and treatment on day 14, 2 mL blood samples were collected by retro orbital puncture in an EDTA-coated tube using all aseptic precautions. The blood sample was kept in an ice bath, and plasma was separated by centrifugation at 1000 rpm for 5 min and stored at −70 °C. Separated plasma samples were used to estimate plasma corticosterone levels, TNF-α, IL-1β and IL-6 expression using an enzyme-linked immunosorbent assay (ELISA) kit. Manufacturer’s instructions, provided with the kit, were followed for evaluation [16].

### 2.9. Plasma Corticosterone

Corticosterone levels in plasma were measured using commercial ELISA kits (Invitrogen) following manufacturer protocols. Wells were loaded with 50 μL calibrators, controls, and samples (excluding blank wells containing negative control provided with kit) in duplicate. A 150 μL conjugate solution containing corticosterone-HRP (horseradish peroxidase) conjugates was added to each well except blanks, followed by 60 min incubation at 37 °C. The solution was thoroughly aspirated and washed using automatic microplate washers (RoBonik) with 5 wash cycles at 350 μL of 1 “x” wash buffer solution per cycle. A 90 μL TMB solution was added per well for 15 min incubation at 37 °C in darkness, then a 50 μL stop solution (1 N HCl) was added in each well with 1–2 min gentle shaking. The color change from blue to yellow was measured at 450 nm.

### 2.10. Qantification of Pro-Inflammatory Cytokines

Pro-inflammatory cytokines (IL-6, IL-1β and TNF-α) levels in serum were measured using a rat-specific ELISA kit (Diaclone). Manufacturer instructions were followed for quantification. To estimate IL-6, IL-1β and TNF-α levels, 100 μL blood serum, controls and diluted standards and 50 µL diluted biotinylated antibodies were added to 96-well plates and incubated at room temperature (18–25 °C) for 3 h. Then, plates were washed thoroughly 3 times with 0.3 mL 1 “x” wash buffer, and 100 μL of diluted streptavidin-HRP was added to each well. Furthermore, plates were sealed and incubated for 30 min at room temperature, followed by thorough washing 3 times with wash buffer. A total of 100 μL of TMB substrate solution was added to each well and incubated for 30 min at room temperature in the dark and stop solution (100 µL of 1N H_2_SO_4_) was subsequently added into each well. Absorbance was measured immediately at 450 nm using an ELISA plate reader [17].

### 2.11. Antioxidant Activity in Rats Brain Tissue Homogenate

After the last stress and treatment on day 14, rats were sacrificed by decapitation and brain tissue was removed, weighed and homogenized in ice-cold 50 mM potassium phosphate buffer (pH 7.4) containing 1 mM EDTA at a 1:10 ratio. Protein concentrations were determined using the Bradford methodology invented in 1976 [29].

#### 2.11.1. Lipid Peroxidation

Lipid peroxidation (LPO) levels were determined by measuring malondialdehyde (MDA) concentration spectrophotometrically, using 1,1,3,3-tetraethoxypropane as standards, following methodology outlined by Ohkawa in 1979 [30]. In brief, 0.2 mL of processed brain homogenate was mixed with 0.2 mL of sodium lauryl sulfate (SLS) (8.1%), 1.5 mL glacial acetic acid (20%) and a 1.5 mL aqueous solution of thiobarbituric acid (TBA) (0.8%). The resulting mixture volume was made up to 4 mL with deionized water, and a sealed tube was placed in a boiling water bath at 95 °C for 60 min. Thereafter, sample tubes were cooled with tap water, and then added 5 mL mixture of n-butanol: pyridine in (15:1 *v*/*v*) ratio and 1 mL of distilled water were added, followed by centrifuging at 2000 g for 5 min. The organic layer was separated out in a 96-well plate (200 µL) for absorbance measurement at 532 nm using a 96-well microplate reader, and its concentration was expressed as n mol/mg protein.

#### 2.11.2. Superoxide Dismutase

Superoxide dismutase (SOD) activity was measured using methodology outlined by Singh in 2020 [31]. All assay components were prepared in PBS (pH 7.4). The reagent preparation involved dissolving 50 mM anhydrous sodium carbonate (Na_2_CO_3_), 0.1 mM EDTA, and 25µM nitro blue tetrazolium (NBT) in 0.1 M PBS (pH 7.4). For analysis, 100 µL of prepared reagent was mixed with 25 µL of hydroxylamine hydrochloride (NH_2_OH·HCl) and 50 µL of supernatant, and absorbance was monitored at 570 nm for 3 min at regular intervals. Enzyme activity units were defined as the enzyme quantity causing 50% inhibition of the reaction rate per minute, with results expressed as units (U) of SOD activity/mg protein [31].

#### 2.11.3. Catalase

Catalase (CAT), an enzyme that catalyzes hydrogen peroxide (H_2_O_2_) decomposition to water and oxygen, was analyzed using the protocol established by Sinha in 1972. The reaction mixture contained 1 mL PBS (0.01 M), 0.1 mL brain tissue homogenate, and 0.4 mL H_2_O_2_ (0.2 M). Prepared reagents were added to a 96-well plate and incubated at 37 °C for 1 min. A 2 mL dichromate/acetic acid solution (containing 5% potassium dichromate (K_2_Cr_2_O_7_) and glacial acetic acid in 1:3 ratio) was added and the mixture was boiled at 100 °C for 10 min. Absorbance was measured at 570 nm and CAT activity was reported as U/min/mg protein [32].

#### 2.11.4. Glutathione (GSH)

Brain glutathione levels were determined using the standard protocol established by Beutler in 1963 with minor modifications. For GSH quantification in brain homogenate, 500 µL of 10% brain homogenate was precipitated using 750 µL precipitation solution containing 5% meta-phosphoric acid, 500 mM NaCl, and 600μM EDTA. Samples were subsequently centrifuged at 5000 rpm at 4 °C for 10 min. The assay mixture comprised 50 µL supernatant combined with 200 µL phosphate solutions and 25 µL of freshly prepared DTNB (0.4% in 0.4 M PBS, pH 7.4). The yellow color was developed then, and its absorbance was read immediately at 412 nm. GSH concentration was expressed as µM/mg of protein [33].

### 2.12. Histopathology of Stomach

The rats were anesthetized with ether and perfused with 150 mL of phosphate-buffered saline (PBS) (0.1 M, pH 7.2) followed by 250 mL of ice-cold 4% paraformaldehyde in PBS for stomach tissue fixation. Tissues were embedded in paraffin blocks and sectioned at 3–5 μm thickness, mounted on gelatin-coated slides, and stained with hematoxylin and eosin (H&E). H&E-stained slides were examined under bright-field microscopy at 100 “x” magnification [20].

### 2.13. Gastric Ulceration Assessment

Post-stress stomachs were removed via greater curvature incisions to measure stress-induced ulcer severity. Discrete ulcers were scored using a magnoscope with histological confirmation. Briefly, the degree and intensity of ulcers were scored as follows: 0 = absence of ulcers; 1 = incomplete superficial mucosal changes without congestion; 2 = necrotic changes and congestion affecting half mucosal thickness; 3 = necrotic changes and congestion involving over two-thirds mucosal thickness; 4 = complete mucosal destruction with significant hemorrhage.

### 2.14. Adrenal Gland and Spleen Weights

After decapitation of each rat, adrenal glands and spleens were removed and weighed. Organ weights were standardized as mg of organ weight per 100 gm of BW [16].

### 2.15. Statistical Analysis

Experimental data are expressed as mean ± SD per group. Data calculations were performed using Microsoft Office Excel 2010 version. Statistical significance was determined using One-way ANOVA (Analysis of variance) with GraphPad Prism (version-10.1.2), followed by a post hoc Dunnett multiple comparison test. Differences were considered statistically significant at *p* < 0.05, *p* < 0.01, *p* < 0.001, and *p* < 0.0001.

## 3. Results

### 3.1. Body Weight, Adrenal Gland and Spleen Weight

**Figure 1:**
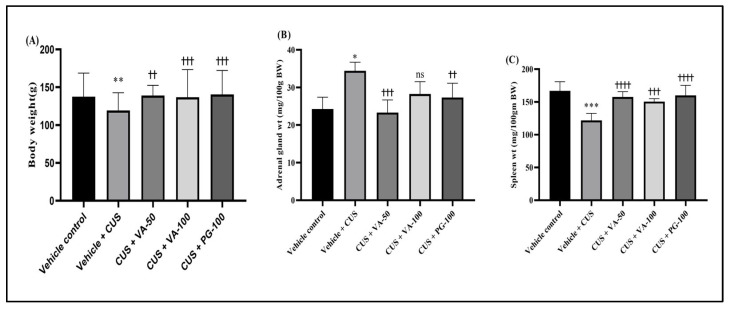
Impact of VA treatment on CUS-induced alteration in (**A**) changes in body weight, (**B**) adrenal gland weight, (**C**) spleen weight in rats. *n* = 6 rats in each group. * *p* < 0.05, ** *p* < 0.01, *** *p* < 0.001 compared to vehicle control; †† *p* < 0.01, ††† *p* < 0.001, †††† *p* < 0.0001 compared to vehicle + CUS. All values expressed as mean ± SD.

Weight reduction constitutes a significant physiological characteristic of animals under stress [11]. The CUS protocol employed in our investigation similarly led to weight reduction in CUS + vehicle rats when compared to vehicle control animals. Throughout the 14-day period, CUS + vehicle-treated rats experienced body weight loss, while weight increases were noted in unstressed vehicle-control animals. Conversely, rats receiving VA and PG treatment demonstrated significantly enhanced weight compared to the CUS + vehicle group. However, VA effect was dose-dependent, and both VA and PG significantly reversed the stress-induced body weight losses (Figure 1A).

Additionally, adrenal gland and spleen weight variations were observed across different experimental groups. CUS caused adrenal hypertrophy and splenic atrophy. Lower VA doses (50 mg/kg/day) and PG (100 mg/kg/day) fully counteracted these stress-induced effects, with no statistically significant differences between treatment groups and the vehicle-treated unstressed control group (Figure 1B,C). Statistically significant beneficial effects of the lowest VA dose on stress-induced pathologies were only partial. These findings confirm that VA has greater potency than standard herbal adaptogen PG.

### 3.2. CUS Induced Behavioral Test

#### 3.2.1. Forced Swimming Test (FST), Tail Suspension Test (TST) and Sexual Behavior Test

**Figure 2:**
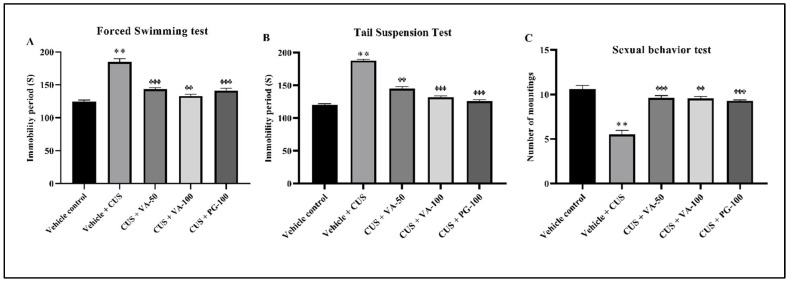
Effect of VA on CUS-induced changes in (**A**) forced swimming test, (**B**) tail suspension test, (**C**) sexual behavior test in rats. *n* = 6 male rats in each group. ** *p* < 0.01 compared to vehicle control; ᶲᶲ *p* < 0.01, ᶲᶲᶲ *p* < 0.001 compared to vehicle + CUS. All values expressed as mean ± SD.

Anxiety and depressive-like behavioral responses in rats serve as robust predictors of stress-related conditions [34], and these behavioral alterations are commonly evaluated using FST, TST, and sexual behavior assessments. FST represents one of the most widely accepted animal models for investigating depressive-like behavior in rodents, where immobility duration serves as a depression indicator. The 14-day CUS protocol resulted in significantly (*p* < 0.01) prolonged immobility time in CUS rats versus control rats in FST (185 vs. 125 s). Figure 2A demonstrates VA impact on immobility duration in the FST model. One-way ANOVA indicated significant differences among treatment groups. Post hoc analysis demonstrated that VA- (50 and 100 mg/kg) and PG-treated groups differed significantly (*p* < 0.01, *p* < 0.001) from the CUS group. VA significantly reduced immobility duration, indicating antidepressant properties. No significant dose-dependent VA effect comparable to PG was observed.

The effects of VA treatment for 14 days were evaluated in rats during a 6 min tail suspension test. Treatment with 50 mg/kg/day of VA resulted in a marked reduction in the duration of immobility. Similarly, administration of VA 100 mg/kg/day and PG 100 mg/kg/day led to a significant reduction in the duration of immobility in rats compared with CUS rats. The results are depicted in Figure 2B.

Compared to non-stressed rats, CUS rats showed significantly diminished sexual behavior. The mean mount frequency in the vehicle-treated CUS group was approximately half that of the vehicle-treated control group (Figure 2C). VA treatments dose-dependently reversed this stress-induced effect, with the observed PG effect magnitude (100 mg/kg/day) being nearly identical to that of VA (100 mg/kg/day).

#### 3.2.2. Light and Dark Chamber Test

**Figure 3:**
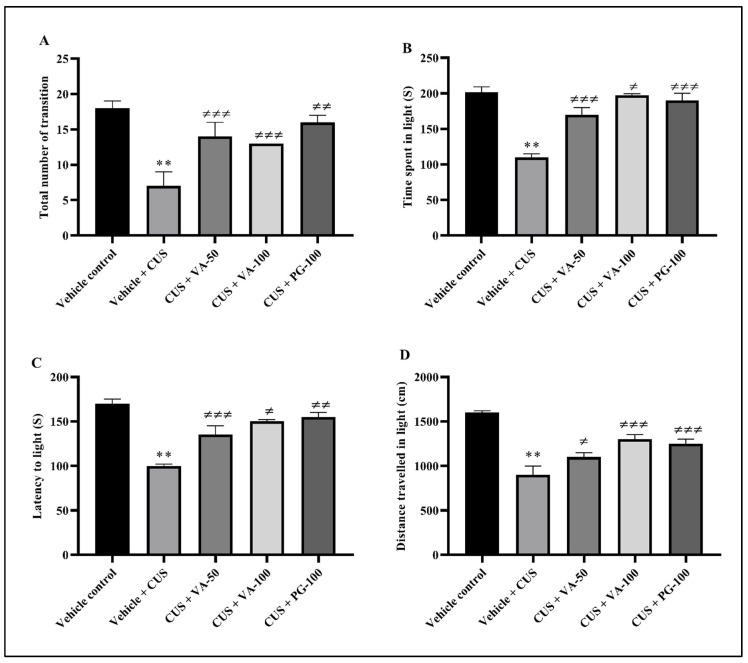
Effect of VA on CUS-induced light and dark chamber test in rats. (**A**) Number of transitions; (**B**) time spent in the light compartment; (**C**) latency time to enter the light compartment; (**D**) traveled distance in the light compartment. *n* = 6 rats in each group, all values are expressed as mean ± SD. Evaluations based on one-way ANOVA followed by Bonferroni multiple test; all groups were compared to the vehicle + CUS group (≠ *p* < 0.05, ≠≠ *p* < 0.01, ≠≠≠ *p* < 0.001). ** *p* < 0.01 compared to vehicle.

Anxiety-like behaviors in VA-treated rats were evaluated through light/dark transition tests. Significant differences were observed in the number of total transitions, time spent in the light chamber, latency to enter the light chamber, and the distance traveled in the light chamber between the VA- and PG-treated groups and CUS + vehicle group rats (Figure 3A–D).

### 3.3. Effect of VA in CUS-Induced Changes in ECG and HRV Signals in Rats

**Figure 4:**
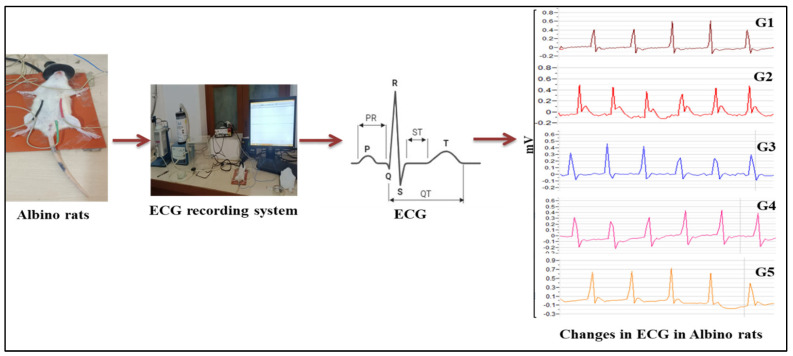
Recording and analysis of an ECG. (G1–G5) represents average ECG pattern of single animals per groups G1: vehicle control, G2: CUS + vehicle, G3 and G4: VA (50 mg/kg and 100 mg/kg), and G5: PG (100 mg/kg)-treated rats, respectively.

ECG serves as a primary diagnostic technique for cardiac activity reflection and provides mechanistic information [35]. ECG and HRV techniques were used to investigate the stress-induced hemodynamic changes in rats (Figure 4). VA treatment significantly prevented the CUS-induced increases in RR interval, heart rate, P amplitude, Q amplitude, R amplitude and ST height in treated group. However, treatment failed to normalize elevated QRS interval, QTc interval, and T peak tend interval induced by CUS. HRV serves as a non-invasive technique for evaluating cardiac autonomic regulations through time-domain, frequency-domain and non-linear domain measurements. All HRV components including Average RR, SD1, Root mean square of successive differences (RMSSD), high frequency (HF), and VLF were reduced in CUS rats and are shown in Box-cum-whisker plots. VA and PG administration in CUS rats significantly prevented the CUS-induced reduction in average and median RR, RMSSD, Low frequency (LF)/HF, HF and VLF, representing sympatho-vagal balance restoration.

**Figure 5:**
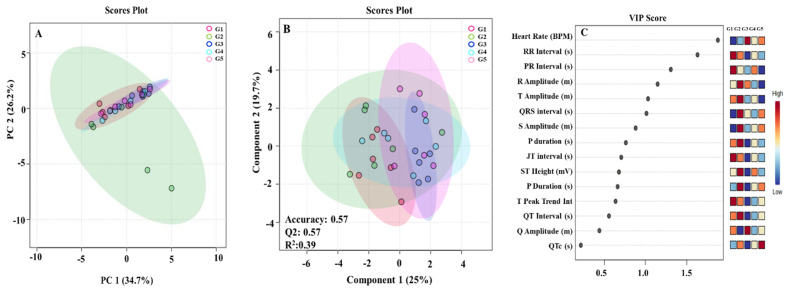

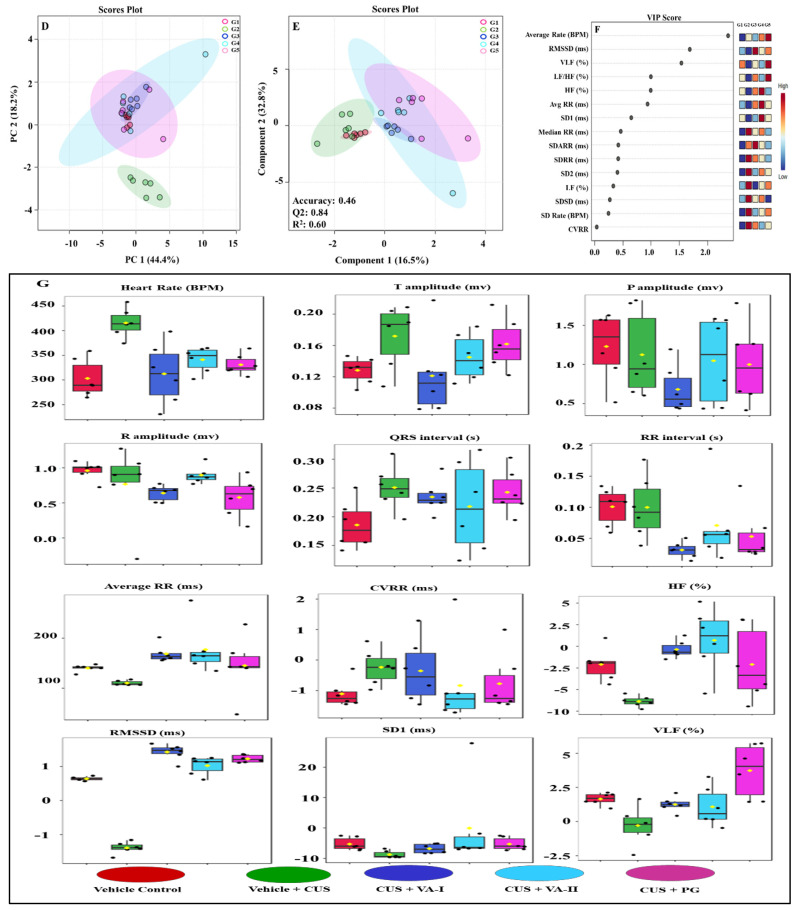
(**A**,**B**,**D**,**E**) represents 2D score plots from PCA and PLS-DA-based analysis for ECG and HRV parameters across treatment groups. Statistical parameters include F-value: 2.2659 and 1.9759; R-squared: 0.26608 and 0.2402; *p*-value: 0.039 and 0.065. Loops indicate the 95% confidence interval for each treatment group. Cross-validation employed by LOOCV methodology with accuracy measured via MetaboAnalyst web server. (**C**,**F**) denotes the VIP score of ECG and HRV parameters across treatment groups. (**G**) Box-cum-whisker plots illustrating quantitative variations in relative signal integrals for ECG and HRV components, demonstrating the effect of VA and PG treatments against CUS-induced stress in rats. ECG parameters exhibit VIP score >0.5 with statistical significance at *p* ≤ 0.05. Box plots represent interquartile ranges (boxes), median values (horizontal lines within boxes), 25th and 75th percentiles (lower and upper box boundaries), and 5th and 95th percentiles (lower and upper whiskers).

Principal Component Analysis (PCA) of ECG and HRV indices suggested therapeutic potential for VA treatment in CUS rats. VA treatment groups showed a compressive shift towards the CUS group, which was further validated by the Partial Least Squares Discriminant Analysis (PLS-DA) model. The PLS-DA model prediction accuracy in ECG and HRV was found to be 0.57 and 0.46, predictive ability (Q2) was found to be 0.57 and 0.84, and R square (R^2^) was found to be 0.39 and 0.60, respectively. The variable importance in projection (VIP) score of PLS-DA model with all important ECG and HRV variables is shown in Figure 5A,B,D,E. In the current study, VIP scores of all ECG parameters exceeded the 0.5 threshold value except Q amplitude and QTc. In addition, eight HRV parameters (Median SDARR, SDRR, SD_2_, LF, SDSD, SD Rate and CVRR) were lower than the threshold value of 0.5VIP score. Similar result patterns were revealed through box plot analysis of ECG and HRV signals (Figure 5G).

### 3.4. Blood Glucose Level

**Figure 6:**
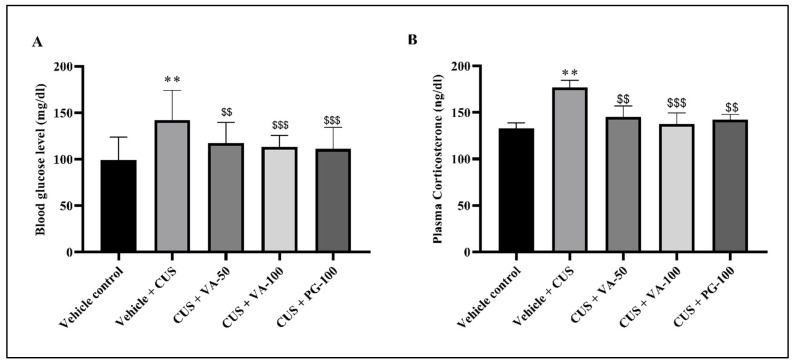
Effect of VA treatment on CUS-induced (**A**) blood glucose level (mg/dL) and (**B**) plasma corticosterone level (ng/dL). *n* = 6 rats in each group. ** *p* < 0.01 in comparison to vehicle; $$ *p* < 0.01, $$$ *p* < 0.001 in comparison to vehicle + CUS. All values are expressed as mean ± SD.

The stress also results in hyperglycemia, which increases the clinical complication of stress-related disorders; if it is not treated, it may be cause mortality in stress-associated diseases. In this study, untreated rats showed a 1.5-fold rise in blood glucose level respective to the CUS. The post-stress treatment of rats with the VA and PG reduced the level of glucose significantly (*p* < 0.01, *p* < 0.001), which was indicated by 0.6 and 0.7-fold glucose level decrease relative to the CUS group. The glucose level decrease was comparable to VA treatment (Figure 6A).

### 3.5. Plasma Corticosterone Level

**Figure 7:**
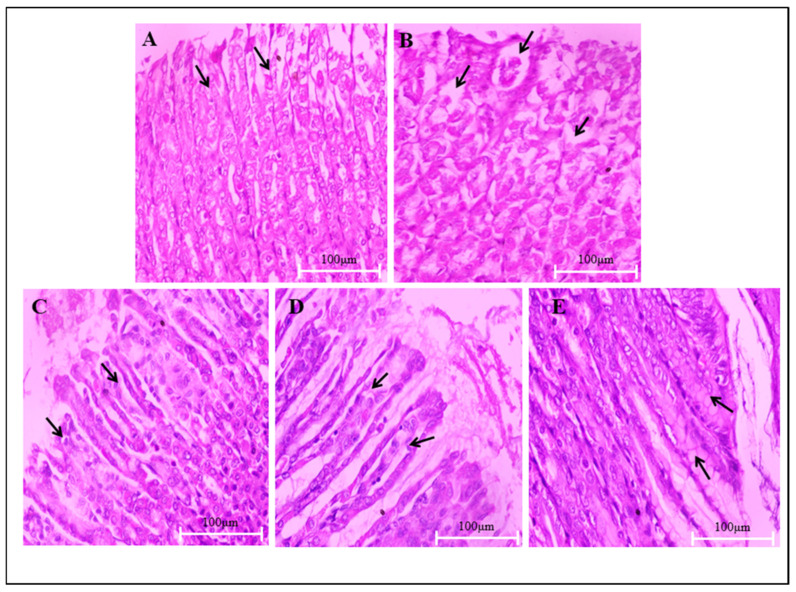
Photomicrographs showing VA effect on the mucous layer in stomach sections of CUS-induced stress (magnification: 40 “x”). (**A**) Stomach of vehicle control rats showing normal mucosal layer, arrows indicating a thick mucus layer. (**B**) Stomach of CUS group, notice the damage of the surface epithelial cells and changes in mucus lining (arrows). (**C**,**D**) Stomach of VA treated groups rats showing recovering of surface epithelial cell (arrows). (**E**) Stomach of PG-treated rats showing normal layer of mucus (arrows).

Elevated plasma corticosterone represents well-known stress markers in rats. CUS significantly augmented plasma corticosterone levels compared to vehicle controls (*p* < 0.01) (Figure 7B). This stress-induced effect was significantly suppressed, however not completely reversed, by VA and PG treatments. Mean plasma corticosterone levels in treatment group were significantly higher than those in the unstressed groups (*p* < 0.01, *p* < 0.001), with clear VA effect dose dependency observed.

### 3.6. Measurement of Gastric Ulceration and Morphological Changes in Stomach

#### 3.6.1. Gastric Ulceration

VA effect on CUS induced gastric ulceration was dose-dependent, exhibiting a modest dose–response relationship, but was not abrupt. However, even after highest doses tested, VA did not provide complete protection against stress-induced ulcers in all animals. Observed PG effects on gastric ulcers were more pronounced than VA higher-dose effects, as mentioned in Table 2.

#### 3.6.2. Histological Evaluation

**Figure 8:**
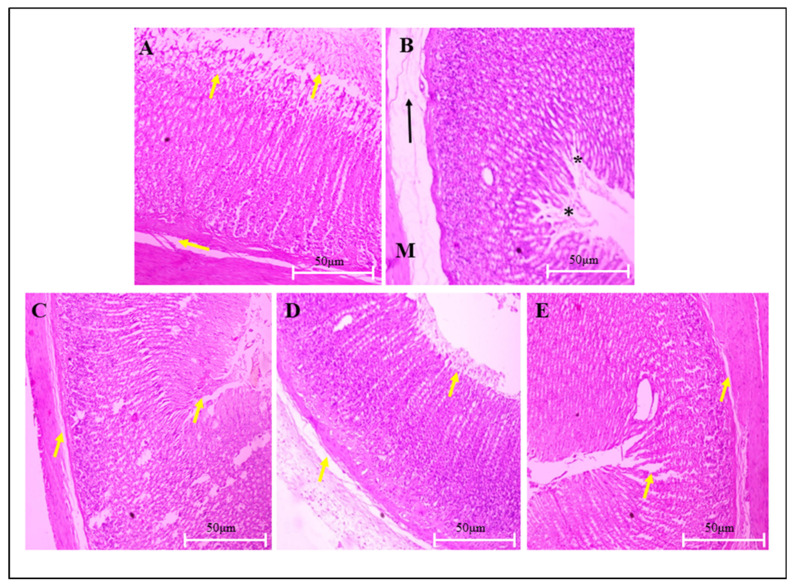
Representative light microscope showing the effect of VA on stomach sections of CUS-induced stressed rats stained with hematoxylin and eosin (H&E) (magnification: 10 “x”). (**A**) Stomach of vehicle control rats showing healthy gastric epithelium with well-organized glandular structure and an appropriate sub-mucosa; (**B**) CUS + vehicle group, * shows damaged mucosal epithelium with interrupted glandular structure, arrow indicates thickness decrease, extensive edema and inflammatory cells infiltration in sub mucosa, and muscular layer thinning (M); (**C**) CUS + VA-50 mg/kg-treated groups showing mild glandular pattern disruption with no submucosal edema; (**D**) rats treated with VA-100 mg/kg + CUS showing nearly normal gastric architecture, with absent epithelial erosion, submucosal edema and leucocytes infiltration, and restoration of normal mucosal, submucosal, and muscular layer thickness; (**E**) rats treated with PG-100 mg/kg + CUS showing nearly normal gastric architecture, with absent epithelial erosion, submucosal edema and leucocytes infiltration, and the restoration of mucosal, submucosal, and muscular layers thickness. Overall, VA treatments show mucosal epithelium retrieval and reorganized glandular structure with improved edema under treatment indicated by yellow arrow.

Microscopic evaluation of CUS-induced histopathological gastric mucosal tissue changes was carried out at 40 “x” and 10 “x” magnifications to observe the effects of VA and PG on mucosal damage in gastric ulcers. Conferring to Figure 7A and Figure 8A, the non-stress vehicle control group revealed intact mucosal epithelium with well-organized layers (mucosa, submucosa, muscularis mucosa and serosa layers). The CUS + vehicle-treated group was characterized by damaged mucosal epithelium, disorganized glandular tissue, and the presence of inflammatory exudates and cellular debris in the ulcerated stomach wall (Figure 7B and Figure 8B). However, the treatment group showed nearly normal gastric glands, with the absent epithelial erosion, submucosal edema, leucocytes infiltration, and normal mucosal, submucosal, and muscular layers thickness restoration. Overall, VA treatment shows mucosal epithelium recovery and reorganized glandular structure with edema improvement under treatment (Figure 7C–E and Figure 8C–E).

### 3.7. Effect of Valeric on Oxidative Stress Markers (LPO, GSH, Catalase, SOD)

**Figure 9:**
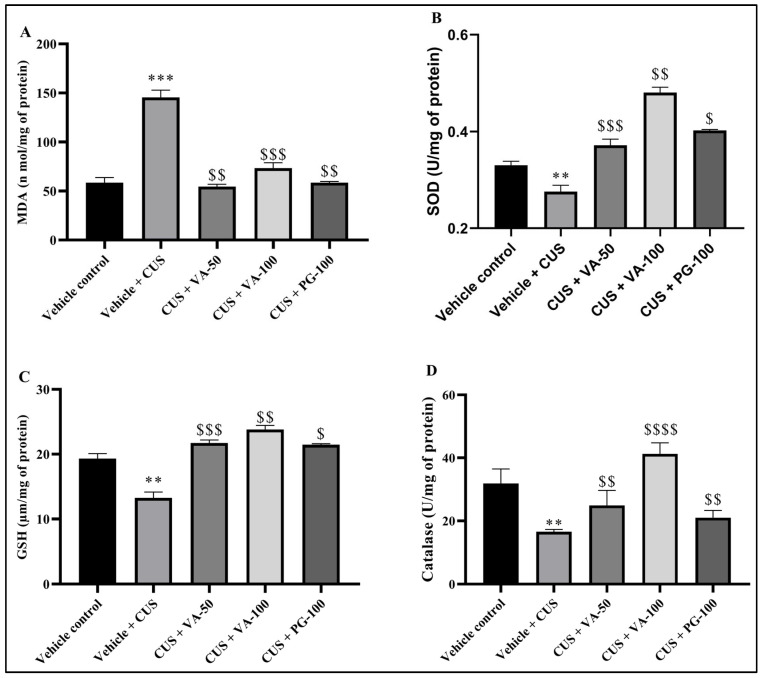
Effect of VA on CUS induced antioxidant parameters; (**A**) LPO, (**B**) GSH, (**C**) SOD, (**D**) Catalase. *n* = 6 in each group. ** *p* < 0.01, *** *p* < 0.001 in comparison to vehicle; $ *p* < 0.05, $$ *p* < 0.01, $$$ *p* < 0.001, $$$$ *p* < 0.001 in comparison to vehicle + CUS. All values are expressed as mean ± SD.

Effect of VA on CUS-induced changes in the antioxidant property was measured by biochemical parameters such as lipid peroxide concentrations (measured by MDA concentrations), glutathione, superoxide dismutase and catalase quantified in brain tissue homogenates. SOD protects cellular components from oxidation by reactive oxygen species (ROS), eventually reducing cellular damage by catalyzing superoxide radical dismutation into molecular oxygen and H_2_O_2_. CAT commonly catalyzes H_2_O_2_ decomposition to water and oxygen. MDA represents oxidative stress markers undergoing thiobarbituric acid condensation producing red fluorescence. CAT is a common enzyme that catalyzes H_2_O_2_ decomposition to water and oxygen. MDA is an oxidative stress marker; it undergoes thiobarbituric acid condensation to produce red fluorescence [31]. MDA levels in the CUS group were significantly increased compared to the corresponding unstressed vehicle-treated group. Meanwhile, GSH, SOD and CAT activities were significantly (*p* < 0.01) decreased compared to the vehicle control group in the brain. Treatment with VA and PG groups reduced the CUS-induced elevated MDA levels (Figure 9A) and restored the reduced GSH, SOD and CAT (Figure 9B–D). Overall, these findings collectively suggest that CUS-induced antioxidant parameters alterations were significantly restored by VA treatments.

### 3.8. Valeric Acid Alter the Serum IL-6, IL-1β and TNF-α

**Figure 10:**
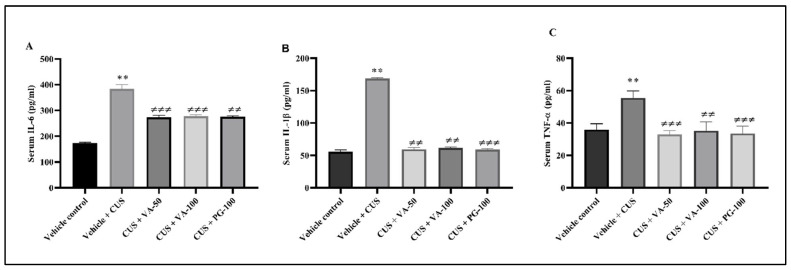
Effect of VA on CUS-induced proinflammatory cytokines expression; (**A**) IL-6, (**B**) IL-1β and (**C**) TNF-α. n = 6 rats in each group. ** *p* < 0.01 compared to vehicle; ≠≠ *p* < 0.01, ≠≠≠ *p* < 0.001 in compared to vehicle + CUS. All values are expressed as mean ± SD.

VA treatment effects on IL-6 levels in CUS rats were quantified in serum after 14 days of CUS exposure. A 2.2-fold IL-6 level increase was observed in serum samples of CUS + vehicle-treated rats compared to the control group (*p* < 0.01). Fourteen days of VA treatment significantly prevented the CUS-induced elevated serum IL-6 levels (*p* < 0.01) in rats. Similarly, PG-treated rats prevent the CUS-induced elevated IL-6 levels. Overall, VA significantly attenuated the CUS induced raised serum IL-6 levels in rats; results are depicted in Figure 10A. The IL-1β level in blood serum was assessed using an ELISA kit. The serum IL-1β level was increased threefold in CUS group rats compared to rats without CUS that were only vehicle-treated (*p* < 0.01). VA treated rats reduce the CUS-induced IL-1β level in blood serum approximately 2.8-fold (Figure 10B). As shown in Figure 10C, TNF-α levels in the blood serum were markedly increased 1.5-fold in CUS group rats compared to vehicle control group rats (*p* < 0.01). Treatment with VA resulted in significant prevention of an increase in TNF-α level in CUS-induced stressed rats.

## 4. Discussion

Stress is a common aspect of daily life, but prolonged chronic stress exposure can have serious health consequences. Long-term chronic stress exposure can shorten lifespan and increase disease risk in susceptible individuals such as people with cardiac disorders, hypertension, metabolic syndrome and obesity, type II diabetes, mental illness and arthritis [35,36]. Homeostatic survival mechanisms in living organisms manage the internal environment to maintain good health. CUS-induced responses can disrupt or imbalance this mechanism, involving neuroendocrine and biochemical processes. Previous studies observed VA has neuroprotective effects in rat models by inhibiting rotenone-induced pro-inflammatory cytokine elevation, oxidative stress, and α-synuclein expression, leading to essential antioxidant enzyme increases [37]. Another study showed that VA has the ability to improv rat learning and memory by reducing the amyloid β1–42 biomarker in aluminum chloride-induced neuronal impairment [38]. VA is a straight-chain alkyl carboxylic acid naturally found in *Valeriana officianilis* and has been used in neurological disorder treatment [18]. PG was selected as a standard drug in this study due to its well-known adaptogenic properties and experimental validation in stress research. As a standard adaptogen, PG has robust anti-stress [16], anxiolytic [39] and neuroprotective action across multiple preclinical models, with particular relevance to stress pathways [40]. Ginsenoside, the principal bioactive constituents of PG, have been shown to protect neuronal tissue from oxidative damage and counteract the detrimental effect of chronic stress on brain structure and function. These effects are mediated by multiple mechanisms, including modulation of the HPA axis, enhancement of endogenous antioxidant defense systems, and suppression of neuroinflammatory pathways [40]. The present study validated a rat model of psychophysiological chronic unpredictable stress. Rats were exposed to 14 days of alternating psychological and physical stressors and subsequently evaluated for psychophysiological stress-related alterations. Consistent with previous reports, CUS produced significant reductions in body weight and increased corticosterone levels, both of which are recognized physiological markers of chronic stress [11]. Treatment with VA effectively reversed the stress-induced loss of body weight.

Chronic psychophysiological stress activates corticotropin-releasing hormone (CRH) pathways, which exert potent anorectic effects. Prolonged stress leads to hyperactivation of CRH-expressing parvocellular neurons in the paraventricular nucleus of the hypothalamus (PVNp), contributing to the sustained reduction in body weight gain observed in CUS-exposed rats. In parallel, hyperactivation of the hypothalamic–pituitary–adrenal (HPA) axis and the sympatho-adrenomedullary system results in elevated corticosterone levels and the development of anxiety- and depressive-like behaviors [35].

Adrenal hypertrophy represents an adaptive response to increased corticosterone demand during chronic stress. VA treatment completely reversed both adrenal hypertrophy and stress-induced splenic atrophy. The presence of gastric ulcers in CUS-exposed rats is consistent with stress-mediated activation of PVNp pathways within the hypothalamus. Stress-induced gastric lesions result from multifactorial pathology, including gastric neutrophil accumulation, inflammatory cytokine production, free radical generation, antioxidant reduction, and mucosal blood flow decrease [16]. VA and PG treatments significantly reduced CUS-induced ulcer incidence and severity.

CUS serves as a widely used depression model in rats. The behavioral despair test (FST and TST) is commonly employed to assess the effectiveness of the CUS paradigm [41,42]. In rats exposed to CUS, the TST often shows increased immobility, reflecting depressive-like behavior. CUS leads to a reduced struggle in the TST, a behavior linked to feelings of helplessness and despair [34,43]. Rats under CUS demonstrate increased immobility in the FST, which indicates behavioral despair. This test is often used to measure the effectiveness of antidepressant treatments, as stressed rats show less activity, implying a depressive-like state [34]. CUS rats have anxiety-like behaviors, such as increased time spent in the dark chamber, which reflects heightened anxiety. This behavior is linked to an altered stress response and higher anxiety levels [44,45]. The hypothalamic–pituitary–gonadal (HPG) axis and the hormonal stress response system, or HPA axis, interact on several levels to influence reproduction; activation of one influences the function of the other, and vice versa. For example, both testosterone and estrogen hormones control the response of the HPA axis. Stress axis activation, particularly chronic or recurring activation, inhibits the release of both testosterone and estrogen [46]. CUS impairs male rat’s sexual behavior, libido, and mounting frequency due to chronic stress on the HPG axis [47]. Previous studies have demonstrated that chronic stress increases anxiety-like behavior and reduces social interaction in rodents. Our finding indicates that chronic stress alters behavioral performance such as locomotor activity and decreases immobility time in the FST, suggesting significant psychophysiological stress-induced behavioral changes [48]. Treatment with VA significantly reduced FST and TST immobility time, comparable to PG effects, suggesting antidepressant-like activity in CUS-induced depression models. Sexual behavior tests further supported VA has antidepressant effects, with dose-dependent reversal of stress-induced mounting reduction. Light-dark chamber tests demonstrated CUS-induced anxiety-like behavior through decreased transitions, reduced light chamber time, shorter light chamber entry latency, and decreased light chamber distance. VA treatment significantly improved these parameters, indicating anxiolytic effects. Overall, VA exhibits both antidepressant and anxiolytic effects in CUS-induced behavioral changes, with dose-dependent efficacy comparable to PG.

Excessive free radical production leads to oxidative damage to the brain [49]. CUS is consistently linked to oxidative stress through an imbalance between the production of ROS and the biological system [50,51]. The brain has high oxygen requirements and peroxide-sensitive lipid abundance makes it particularly ROS-vulnerable. Excessive ROS levels disrupt cellular function and integrity, causing oxidative stress that leads to enzyme inactivation, protein oxidation, DNA damage, lipid peroxidation, and protein denaturation. Previous studies confirmed that oxidative stress plays an important role in neurodegenerative diseases, including depression, memory impairment, anxiety, Alzheimer’s disease and Parkinson’s disease [52,53]. ROS and free radicals drive oxidative stress through GSH and SOD depletion, which normally detoxify ROS and protect against tissue damage [54,55]. The end product of lipid peroxidation (MDA) was higher in CUS rat brains than VA treated groups, indicating increased oxidative damage. Endogenous antioxidant defenses normally limit the ROS-induced oxidative stress through dismutation of SOD by superoxide radicals to H_2_O_2_, catalase conversion to H_2_O, and GSH catalyzed glutathione reaction with H_2_O_2_ [56]. CUS decreased brain GSH, SOD, and catalase activities, suggesting that CUS causes brain oxidative stress by down-regulating GSH and catalase.

Corticosterone acts as the primary stress hormone in rodents [57]. Stress-induced pro-inflammatory cytokine changes initiate psychiatric disorder (depression), autoimmune diseases, and cancer [58]. A number of studies suggest that stressful experiences can trigger inflammatory responses not only in the brain but also throughout the peripheral tissues [59]. Stress activates the HPA axis through hypothalamic CRH secretion, which typically inhibits immunological response by releasing GCs from the adrenal glands [11]. Increased corticosteroid secretion in stress contributes to metabolic disturbances and leads to glucose intolerance in chronic stressed rats, who exhibit elevated blood glucose levels [60]. In our study, administration of VA and PG resulted in the restoration of these heightened glucose levels. Our data confirm that CUS exposed elevated proinflammatory cytokines (IL-6, IL-1β and TNF-α) level restored in rats, consistent with previously reported findings [61,62]. Stressed rats showed higher cytokine levels compared to non-stressed rats. VA treatment restored altered pro-inflammatory cytokine levels throughout treatment.

During stressful situations, the body triggers the release of adrenaline and cortisol, which stimulate sympathetic nervous system commonly referred as the “fight or flight” mechanism. This activation elevates both heart rate and blood pressure, readying the organism for responsive action [3]. Electrocardiography evaluates cardiac electrical activity and myocardial structure/function in both humans and rodents, though action potential characteristics differ [63]. CUS rats showed increased RR interval, heart rate, P amplitude, Q amplitude, R amplitude, and ST height, plus decreased PR interval, S amplitude, and QT interval, indicating electrical conduction system disruption. These sinus rhythm variations link to cardiac anatomical and functional modifications. Decreased PR intervals indicate ventricular pre-excitation, increased T amplitude associates with cardiac abnormalities, and elevated ST segments frequently link with myocardial infarction [35]. In the present study, VA-treated CUS rats showed prevention of CUS-induced ECG alterations, preventing increases in PR interval, S amplitude, and QT interval. However, HRV provides non-invasive cardiac autonomic assessment. Time domain indices RMSSD and SD1 were reduced in chronically stressed rats, indicating diminished parasympathetic (vagal) activity [64,65]. Frequency domain measures include LF, HF, and LF/HF ratios. HF reflects parasympathetic function, LF represents combined sympathetic and parasympathetic activity, while LF/HF ratio indicates autonomic balance [66]. Decreased LF/HF and HF values in CUS rats demonstrate sympatho-vagal imbalance and reduced vagal tone. Moreover, VA and PG treatment prevented most CUS-induced ECG changes, including elevated RR intervals, heart rate, P, Q, R amplitudes, and ST height, though S amplitude and QRS interval increases persisted. VA treatment restored sympatho-vagal balance, normalizing HRV parameters (average and median RR, RMSSD, SDARR, LF/HF, VLF). Thus, VA demonstrated protective effects against CUS-induced heart function abnormalities and autonomic heart flow.

## 5. Conclusions

Our study validated an in vivo rodent model of chronic unpredictable stress (CUS) and demonstrated that valeric acid has the potential to counteract stress-induced physiological and behavioral disturbances. VA administration significantly improved key stress biomarkers, including corticosterone and glucose levels, indicating a restoration of HPA axis function. Behavioral analyses showed marked reductions in immobility time in the forced swimming and tail suspension tests, along with improved performance in the sexual behavior test. VA also attenuated depression-like behavior in the light–dark chamber test. Inflammation, a central mediator of stress pathology, was notably suppressed following VA treatment. These findings collectively suggest that VA provides protective effects against chronic unpredictable stress. Although the present results highlight the anti-stress potential of VA, its broader relevance—particularly in stress-associated disorders—remains to be clarified. Future research should include long-term studies, detailed investigation of underlying molecular mechanisms, and validation across additional experimental models. Clinical studies will ultimately be required to establish the safety, efficacy, and translational potential of VA in humans.

## Figures and Tables

**Table 1:** Schedule of stressors applied for 14 days.

Days of Stressor	Time (h)	Stressor Applied	Duration
1	10:00	Cold restraint	3 h
	14:00	Water deprivation	20 h
2	10:00	Tail pinching	5 min
	16:00	Wet cage bedding	15 h
3	07:00	Isolation	12 h
	19:00	Day night reversal	15 h
4	10:00	Forced swimming	30 min
	14:00	Fasting (food deprivation)	20 h
5	10:00	Restraint	3 h
	16:00	Tail pinching	5 min
6	10:00	Cold restraint	3 h
	14:00	Water deprivation	20 h
7	10:00	Tail pinching	5 min
	16:00	Wet cage bedding	15 h
8	07:00	Isolation	12 h
	19:00	Day night reversal	15 h
9	10:00	Forced swimming	30 min
	14:00	Fasting (food deprivation)	20 h
10	10:00	Restraint	3 h
	16:00	Tail pinching	5 min
11	10:00	Cold restraint	3 h
	14:00	Water deprivation	20 h
12	10:00	Tail pinching	5 min
	16:00	Wet cage bedding	15 h
13	10:00	Forced swimming	30 min
	14:00	Fasting (food deprivation)	20 h
14	10:00	Restraint	3 h
	16:00	Tail pinching	5 min

**Table 2:** Effect of valeric acid on CUS-induced gastric ulceration and morphological changes in rats.

Treatment	Dose (mg/kg/p.o)	Mean Ulcer Score	Severity of Ulcer
Vehicle control	0.3% CMC	0.0 ± 0.0	0.0 ± 0.0
Vehicle + CUS	0.3% CMC	14.83 ± 1.52 **	33.50 ± 4.50 **
CUS + VA	50	8.66 ± 1.05 *	15.12 ± 2.25 *
CUS + VA	100	7.14 ± 0.98 *	10.65 ± 1.96 *
CUS + PG	100	5.11 ± 1.11 *	8.75 ± 1.85 *

CUS = Chronic unpredictable stress, VA = Valeric acid, PG = *Panax ginseng*, *n* = 6 rats in each group. ** *p* < 0.01 compared to vehicle control; * *p* < 0.05 compared to Vehicle + CUS. Statistical analysis performed using Kruskal–Wallis test (non-parametric test) followed by Dunn’s post hoc test with Bonferroni correction for multiple comparison.

## Data Availability

The data that support the findings of this study are available in the article.

## Abbreviations

The following abbreviations are used in this manuscript:

ACTH	Adrenocorticotropic hormone
ANS	Autonomic nervous system
CAT	Catalase
CUS	Chronic Unpredictable Stress
ECG	Electrocardiogram
ELISA	Enzyme-linked immunosorbent assay
FST	Forced swimming test
HRV	Heart rate variability
H&E	Hematoxylin and eosin
HPA	Hypothalamic–Pituitary–Adrenal axis
LPO	Lipid peroxidation
MDA	Malondialdehyde
PG	Panax ginseng
SOD	Superoxide dismutase
TST	Tail suspension test
VA	Valeric acid
